# UMP-CMP kinase 2 gene expression in macrophages is dependent on the IRF3-IFNAR signaling axis

**DOI:** 10.1371/journal.pone.0258989

**Published:** 2021-10-27

**Authors:** Hera Kim, Yashwanth Subbannayya, Fiachra Humphries, Astrid Skejsol, Sneha M. Pinto, Miriam Giambelluca, Terje Espevik, Katherine A. Fitzgerald, Richard K. Kandasamy

**Affiliations:** 1 Centre of Molecular Inflammation Research (CEMIR), and Department of Clinical and Molecular Medicine (IKOM), Norwegian University of Science and Technology, Trondheim, Norway; 2 Program in Innate Immunity, University of Massachusetts Medical School, Worcester, MA, United States of America; 3 College of Medicine, Mohammed Bin Rashid University of Medicine and Health Sciences, Dubai, UAE; University of Nebraska-Lincoln, UNITED STATES

## Abstract

Toll-like receptors (TLRs) are highly-conserved pattern recognition receptors that mediate innate immune responses to invading pathogens and endogenous danger signals released from damaged and dying cells. Activation of TLRs trigger downstream signaling cascades, that culminate in the activation of interferon regulatory factors (IRFs), which subsequently leads to type I interferon (IFN) response. In the current study, we sought to expand the scope of gene expression changes in THP1-derived macrophages upon TLR4 activation and to identify interferon-stimulated genes. RNA-seq analysis led to the identification of several known and novel differentially expressed genes, including CMPK2, particularly in association with type I IFN signaling. We performed an in-depth characterization of CMPK2 expression, a nucleoside monophosphate kinase that supplies intracellular UTP/CTP for nucleic acid synthesis in response to type I IFN signaling in macrophages. CMPK2 was significantly induced at both RNA and protein levels upon stimulation with TLR4 ligand—LPS and TLR3 ligand—Poly (I:C). Confocal microscopy and subcellular fractionation indicated CMPK2 localization in both cytoplasm and mitochondria of THP-1 macrophages. Furthermore, neutralizing antibody-based inhibition of IFNAR receptor in THP-1 cells and BMDMs derived from IFNAR KO and IRF3 KO knockout mice further revealed that CMPK2 expression is dependent on LPS/Poly (I:C) mediated IRF3- type I interferon signaling. In summary, our findings suggest that CMPK2 is a potential interferon-stimulated gene in THP-1 macrophages and that CMPK2 may facilitate IRF3- type I IFN-dependent anti-bacterial and anti-viral roles.

## Introduction

The innate immune response provides the first line of defense against invading pathogens. It utilizes several pattern recognition receptors (PRRs), such as Toll-like receptors (TLRs), C-type lectin receptors (CLRs), nucleotide-binding oligomerization domain (NOD)-like receptors, RIG-I-like receptors (RLRs), and AIM2-like receptors (ALRs) to detect conserved molecular structures of invading microbes called pathogen-associated molecular patterns (PAMPs) [1]. Among the various PRRs, TLRs are the major and most extensively studied class that plays a crucial role in initiating innate immune responses [2]. The PAMP lipopolysaccharide (LPS), a major outer membrane component of Gram-negative bacteria, binds to TLR4 and triggers downstream signaling events that culminate in the production of numerous genes that are critical for systematic inflammatory response, including chemokines, pro-inflammatory cytokines (such as IL-1α/β, IL-18, IL-6, and TNFα) and type I interferons (IFNs) [1, 3].

Production of type I IFNs such as IFN-α and IFN-β, relies on the phosphorylation of transcription factors, namely IFN regulatory factors (IRFs), which is mediated by TANK-binding kinase 1 (TBK1) and inhibitor-κB kinase ε (IKKε) upon TLR activation [4, 5]. Both IFN-α and IFN-β can be secreted and, in turn, bind to interferon receptors (IFNARs) in both an autocrine and paracrine manner. This binding leads to the activation of the Janus kinase (JAK)-signal transducer and activator of transcription (STAT) signaling pathway, which results in direct anti-bacterial and anti-viral effects of type I IFNs and transcriptional activation of hundreds of interferon-stimulated genes (ISGs) [6–8]. Studies have demonstrated the vital role of ISGs in restricting intracellular replication of viruses and bacteria by directly targeting pathways and stages of the pathogen life cycle [9, 10]. For example, the murine myxovirus resistance 1 (Mx1) broadly inhibits a wide range of viruses by trapping incoming virus particles and uncoating the viral ribonucleocapsids [11]. ISG15, on the other hand, modifies host proteins and thereby inhibits viral replication or virus release. Also, secreted ISG15 acts as a cytokine and modulates immune responses [12]. Viperin, also known as RSAD2, has been demonstrated to possess anti-viral activity against a broad range of RNA and DNA viruses [13]. Recent studies have suggested that Viperin inhibits RNA replication of hepatitis C virus (HCV) or HIV-1 and influenza A virus particle release [14–17]. A small number of ISGs have been demonstrated to possess anti-bacterial effects. Members of the interferon (IFN)-induced transmembrane (IFITM) protein family have been shown to restrict intracellular growth of *Mycobacterium tuberculosis* (MTb) probably through their mediation in endosomal maturation [18]. The ISG viperin has been shown to inhibit the growth of *Shigella flexneri*, an intracellular bacteria, *in vitro* [19]. A cell-based screen with over 350 human genes identified six ISGs, including *PKRD2*, *UNC93B1*, *MYD88*, *AQP9*, *MAP3K14*, *and TRIM14*, that robustly inhibited *Listeria monocytogenes* infection [20]. These studies suggest the importance of ISGs that can be potentially modulated to regulate interferon responses. Additionally, dysregulated interferon signaling has been implicated in several diseases, including autoimmune diseases such as Systemic Lupus Erythematosus (SLE), Systemic Sclerosis (SS), Rheumatoid arthritis (RA) [21, 22], and cancers [23–25]. Owing to their role in several diseases, the characterization of ISGs is essential. Although several ISGs have been identified, detailed characterization of their roles in modulating immune responses is yet to be deciphered.

PMA differentiated THP-1 cells (a human monocytic leukemia cell line) are valuable models that resemble native monocyte-derived macrophages in various aspects [26, 27]. In the current study, we performed RNA-Seq analysis of LPS-stimulated THP-1 macrophage-like cells to identify potential interferon-inducible genes. Among differentially expressed genes identified, *CMPK2*, a nucleoside monophosphate kinase that supplies intracellular UTP/CTP for nucleic acid synthesis, was observed to be highly inducible. The expression dynamics were found to be similar to several well-known ISGs. However, the role of CMPK2 in modulating type I IFN signaling responses is not well described. Thus, we aimed to characterize CMPK2 gene regulation in response to different TLR stimuli. Here, we show that expression of CMPK2 is induced explicitly by LPS and Poly (I:C), localized in both cytoplasm and mitochondria, and its expression is IRF3-type I-IFN dependent. Overall, our results may provide insights into the anti-viral and anti-bacterial mechanisms of CMPK2 and guide future therapies for interferon-related inflammatory diseases.

## Material and methods

### Reagents

The following ligands were used: Pam3CSK4 (TLR1/2; tlrl-pms; Invivogen, 200 ng/ml), FSL1 (TLR2/6; tlrl-fsl; Invivogen, 100 ng/ml), Poly (I:C) (TLR3; vac-pic; Invivogen, 10 μg /ml), LPS K12 (TLR4; tlrl-eklps; Invivogen, 200 ng/ml), Flagellin (TLR5; tlrl-stfla; Invivogen, 100 ng/ml), R837 (TLR7; tlrl-imqs; Invivogen, 10 μg/ml), R848 (TLR7/8; tlrl-r848; Invivogen, 100 ng/ml), CL075 (TLR8; tlrl-c75; Invivogen, 5 μg/ml), and CpG2006 (TLR9; tlrl-2006; Invivogen, 10 μM), and human interferon-beta 1a (100U/ml) was purchased from PeproTech GmbH (#300–02BC; Germany), 2’3’-cGAMP (InvivoGen, 1ug/ml), Lipofectamine 3000 (Invitrogen), diABZI STING agonist-1 (MedChemExpress, 1μM), Sendai Virus (Cantell Strain) (Charles River, 10 HA units/ml), and HSV-1 (MOI = 1), infected as previously described [28].

### Mice

All animal experiments were approved by the Institutional Animal Care Use Committees at the University of Massachusetts Medical School. Animal were kept in specific pathogen free (SPF) environment. Mice were sacrificed after euthanizing using CO2. For isolation of BMDMs, tibias and femurs were removed from wild-type mice and bone marrow was flushed with complete DMEM-medium. Cells were plated in a medium containing 20% (v/v) conditioned medium of L929 mouse fibroblasts cultured for 7 days at 37°C in a humidified atmosphere of 5% CO_2_. The medium was replaced every 3 days. Irf3-/- and Ifnar1 -/- mice were used as previously described [29].

### Cell culture and differentiation

Human THP-1 monocytic cells (ATCC) were grown in RPMI 1640 (Sigma-Aldrich) supplemented with 10% heat-activated fetal calf serum (FCS), 2 mM L-glutamine, 100 nM penicillin/streptomycin (Thermo Fisher Scientific), and 50 μM *β*-mercaptoethanol (Sigma-Aldrich) and cultured at 37°C in a 5% CO_2_ atmosphere. THP-1 cells were differentiated with 50 ng/ml phorbol-12-myristate-13-acetate (PMA; Sigma-Aldrich, Darmstadt, Germany) overnight (16 hours) and rested in a PMA-free medium for 48 hours before further experiments [30].

### RNA isolation and cDNA library preparation for transcriptome sequencing (RNA-seq)

Total RNA was extracted using QIAzol (79306; Qiagen, Germany), followed by DNAse digestion (Qiagen, Germany) treatment on the RNeasy Mini columns, according to the manufacturer’s protocol. The amount of total RNA was quantified using the Qubit 2.0 Fluorometric Quantitation system (Thermo Fisher Scientific, Waltham, MA, USA), and the RNA integrity number (RIN) was determined using the Experion Automated Electrophoresis System (Bio-Rad, Hercules, CA, USA). RNA-seq libraries were prepared with the TruSeq Stranded mRNA LT sample preparation kit (Illumina, San Diego, CA, USA) using Sciclone and Zephyr liquid handling workstations (PerkinElmer, Waltham, MA, USA) for pre- and post-PCR steps, respectively. Library concentrations were quantified with the Qubit 2.0 Fluorometric Quantitation system (Life Technologies, Carlsbad, CA, USA). The size distribution was assessed using the Experion Automated Electrophoresis System (Bio-Rad, Hercules, CA, USA). For sequencing, samples were diluted and pooled into NGS libraries in equimolar amounts.

### Next-generation sequencing and raw data acquisition

Expression profiling libraries were sequenced on HiSeq 3000/4000 instruments (Illumina, San Diego, CA, USA) following a 50-base-pair, single-end recipe. Raw data acquisition (HiSeq Control Software, HCS, HD 3.4.0.38) and base calling (Real-Time Analysis Software, RTA, 2.7.7) were performed on-instrument, while the subsequent raw data processing off the instruments involved two custom programs (https://github.com/DanieleBarreca/picard/) based on Picard tools (2.19.2) (https://broadinstitute.github.io/picard/). In the first step, base calls were converted into lane-specific, multiplexed, unaligned BAM files suitable for long-term archival (IlluminaBasecallsToMultiplexSam, 2.19.2-CeMM). In the second step, archive BAM files were demultiplexed into sample-specific, unaligned BAM files (IlluminaSamDemux, 2.19.2-CeMM).

### Transcriptome analysis

NGS reads were mapped to the Genome Reference Consortium GRCh38 assembly via “Spliced Transcripts Alignment to a Reference” (STAR) [31] utilizing the “basic” Ensembl transcript annotation from version e100 (April 2020) as reference transcriptome. Since the hg38 assembly flavor of the UCSC Genome Browser was preferred for downstream data processing with Bioconductor packages for entirely technical reasons, Ensembl transcript annotation had to be adjusted to UCSC Genome Browser sequence region names. STAR was run with options recommended by the ENCODE project. Aligned NGS reads overlapping Ensembl transcript features were counted with the Bioconductor (3.11) GenomicAlignments (1.24.0) package via the summarizeOverlaps function in Union mode, taking into account that the Illumina TruSeq stranded mRNA protocol leads to the sequencing of the second strand so that all reads needed inverting before counting. Transcript-level counts were aggregated to gene-level counts and the Bioconductor DESeq2 (1.28.1) package (https://bioconductor.org/packages/release/bioc/html/DESeq2.html) [32] was used to test for differential expression based on a model using the negative binomial distribution.

### Bioinformatics analysis

The initial exploratory analysis included principal component analysis (PCA), multidimensional scaling (MDS), sample distance and expression heatmap plots, all annotated with variables used in the expression modeling (ggplot2, 3.3.2, https://ggplot2.tidyverse.org [33], and Bioconductor ComplexHeatmap,2.4.3, https://bioconductor.org/packages/release/bioc/html/ComplexHeatmap.html), as well as volcano plots (Bioconductor EnhancedVolcano, 1.6.0, https://bioconductor.org/packages/release/bioc/html/EnhancedVolcano.html). Biologically meaningful results were extracted from the model, log2-fold values were shrunk with the CRAN ashr (2.2.-47) package [34], while two-tailed p-values obtained from Wald testing were adjusted with the Bioconductor Independent Hypothesis Weighting (IHW, 1.16.0) package [35]. The resulting gene lists were annotated, filtered for significantly differentially up- and down-regulated genes, and independently subjected to gene set enrichment analysis (Enrichr) (https://amp.pharm.mssm.edu/Enrichr/) [36]. The list of DEGs was compared with genesets obtained from the Molecular Signatures Database (MSigDB, v. 7.0, https://www.gsea-msigdb.org/gsea/msigdb). These genesets included Interferon responsive genes, response to interferon-alpha, beta, and gamma, transcription factors, inflammasome markers, and cytokines and chemokines

### Confocal imaging

Differentiated THP-1 cells were seeded in 24-well glass-bottom plates (MatTek Corporation) and fixed with 2% paraformaldehyde (PFA) as previously described [37]. Cells were then permeabilized using PEM buffer (80 mM K-Pipes, pH 6.8, 5 mM EGTA, 1 mM MgCl_2_, and 0.05% saponin) and quenched in 50 mM NH_4_Cl with 0.05% saponin. After blocking the cells in PBS with 10% human serum and 0.05% saponin, the following primary antibodies: TYKi Antibody (Novus; NBP1-80653) and normal rabbit IgG (sc-2027) (diluted to 2 μg/mL in 1% serum) were added and incubated at 4°C overnight. The next day, cells were washed, and a specific secondary antibody, Alexa Fluor® 488 Chicken anti-Rabbit (Invivogen; A-21441) was added at a concentration of 1 μg/mL in 1% serum in PBS. Cells were then washed again and incubated with Tom20 Antibody (F-10) Alexa Fluor® 546 (Santa Cruz Biotechnology). Lastly, Hoechst (Thermo; 62249) was used for nuclear staining. Confocal imaging was performed on a Leica TCS SP8 (Leica Microsystems) equipped with a HC plan-apochromat 63×/1.4 CS2 oil-immersion objective and the LAS X software, using 488 nm, and 561 nm white laser lines and the 405 nm laser for detection. 12-bit raw imaging data from individual Z-stacks from each channel was used to acquire 3D data. The Bitplane IMARIS software was used for the analysis of colocalization between channels. For each channel, an individual threshold was selected and maintained for all processed samples. 5 different image acquisitions were obtained for each condition. The fraction of CMPK2 colocalized with TOM20 in the 3D volume was calculated and shown as Mander’s coefficient above the threshold. Surface rendering by channel masking was also done in IMARIS.

### Mitochondrial fractionation

Isolation of mitochondrial and cytoplasmic fractions was performed using Human Mitochondria Isolation Kit (Miltenyi Biotec, Germany). Briefly, THP-1 cells were stimulated with LPS (200ng/ml, tlrl-eklps; Invivogen) and Poly (I:C) (10μg/ml, vac-pic; Invivogen) for 24 hours following differentiation and then lysed with 1ml of ice-cold lysis buffer (provided with the kit) containing cOmplete™, Mini, EDTA-free Protease Inhibitor Cocktail (Roche, Mannheim, Germany) and PhosSTOP (Sigma, Darmstadt, Germany). Subsequently, cells were homogenized with a 26.5G syringe 20 times on the ice. The mitochondria were labeled with anti-TOM22 antibodies (Provided in the kit) and isolated according to the Miltenyi Biotec protocol. The supernatant cytosolic fraction was also collected during the magnetic separation process and stored at– 80°C until further use.

### Blocking IFNAR receptors

PMA-differentiated THP-1 cells were preincubated with 2.5 μg/ml Anti-Interferon-α/β Receptor Chain 2 Antibody, clone MMHAR-2 (MAB1155; EMD Millipore, Germany) for 30 minutes at 37°C before stimulation. As a control, Ultra-LEAF™ Purified Mouse IgG2a, κ Isotype MOPC-173 (400224; BioLegend, England) was used. After incubation, cells were stimulated for different time periods and lysed using RIPA buffer for protein extraction.

### RNA isolation and quantitative real-time PCR (qRT-PCR)

Total RNA was extracted using QIAzol (79306; Qiagen, Germany), followed by DNAse digestion (Qiagen, Germany) treatment on the RNeasy Mini columns, according to the manufacturer’s protocol, and cDNA was prepared using the High-Capacity RNA-to-cDNA™ Kit (Applied Biosystems, Vilnius, Lithuania) for RT-qPCR. qPCR was performed on cDNA using the PerfeCTa qPCR FastMix (Quanta Biosciences, Maryland, United States) in duplicates on a StepOne Plus Real-Time PCR cycler (Thermo Fisher Scientific, Germany). The following TaqMan Gene expression Assay (Applied Biosystems, Bleiswijk, Netherlands) were used: CMPK2 (Hs01013364_m1), CMPK1 (Hs00179619_m1), MX1 (Hs00895608_m1), and ISG15 (Hs01921425_s1). Cycle thresholds (Ct) of target genes were normalized to Ct of Eukaryotic18S (VIC™/TAMRA™ probe, 4319413E, Life Technologies, United States). The results are presented as relative expression compared to the control, non-treated samples. Relative expression values were calculated using the Pfaffl’s mathematical model [38].

### Cell lysis and Western blotting

Cells were lysed using 1× RIPA lysis buffer (150 mM NaCl, 50 mM Tris-HCl, pH 7.5, 1% Triton X-100, 5 mM EDTA, protease inhibitors, and phosphatase inhibitors). Protein concentrations were measured by BCA assay (Pierce, Waltham, MA), and samples were resolved on NuPAGE™ Bis-Tris gels (Invitrogen, Bleiswijk, Netherlands) with 1 x MOPS buffer (Invitrogen, Bleiswijk, Netherlands) followed by transfer on nitrocellulose membranes, using the iBlot12 Gel Transfer Device (Invitrogen, California, United States). After washing the membranes in TBS-T (Tris Buffered Saline with 0.1% Tween-X100), blocking of membranes was carried out with 5% BSA for 1hour and then probed with the following primary antibodies overnight at 4°C under gentle agitation: anti-β-tubulin (Abcam, ab15568, Cambridgeshire, England), CMPK1 Monoclonal Antibody (OTI1H8; MA5-26124, Invitrogen), and TYKi Antibody (Novus; NBP1-80653, Oxfordshire, England) GAPDH (Abcam, ab8245, Amsterdam, Netherlands), Anti-COX IV-mitochondrial loading control (Abcam, ab16056, Amsterdam, Netherlands), Phospho-Stat1 (Tyr701) (58D6; CST-#9167, Cell Signaling Technology, Leiden, Netherlands), Phospho-TBK1/NAK (Ser172) (D52C2; CST-#5483, Cell Signaling Technology, Leiden, Netherlands), MX1 (D3W7I; CST-#37849, Cell Signaling Technology, Leiden, Netherlands), and ISG15 (CST-#2743, Cell Signaling Technology, Leiden, Netherlands). Membranes were washed with TBS-T and incubated with respective secondary antibodies (HRP-conjugated, DAKO) at room temperature for 1 hour. The blots were developed using SuperSignal West Femto Substrate (Thermo Scientific, Illinois, United States) and captured with LI-COR Odyssey system (LI-COR Biosciences, Lincoln, NE, USA). The band intensity was quantified using ImageJ analysis software.

## Results

### Temporal transcriptomic analysis of LPS-stimulated THP-1 macrophages identifies markers of TLR4 activation

**Fig 1:**
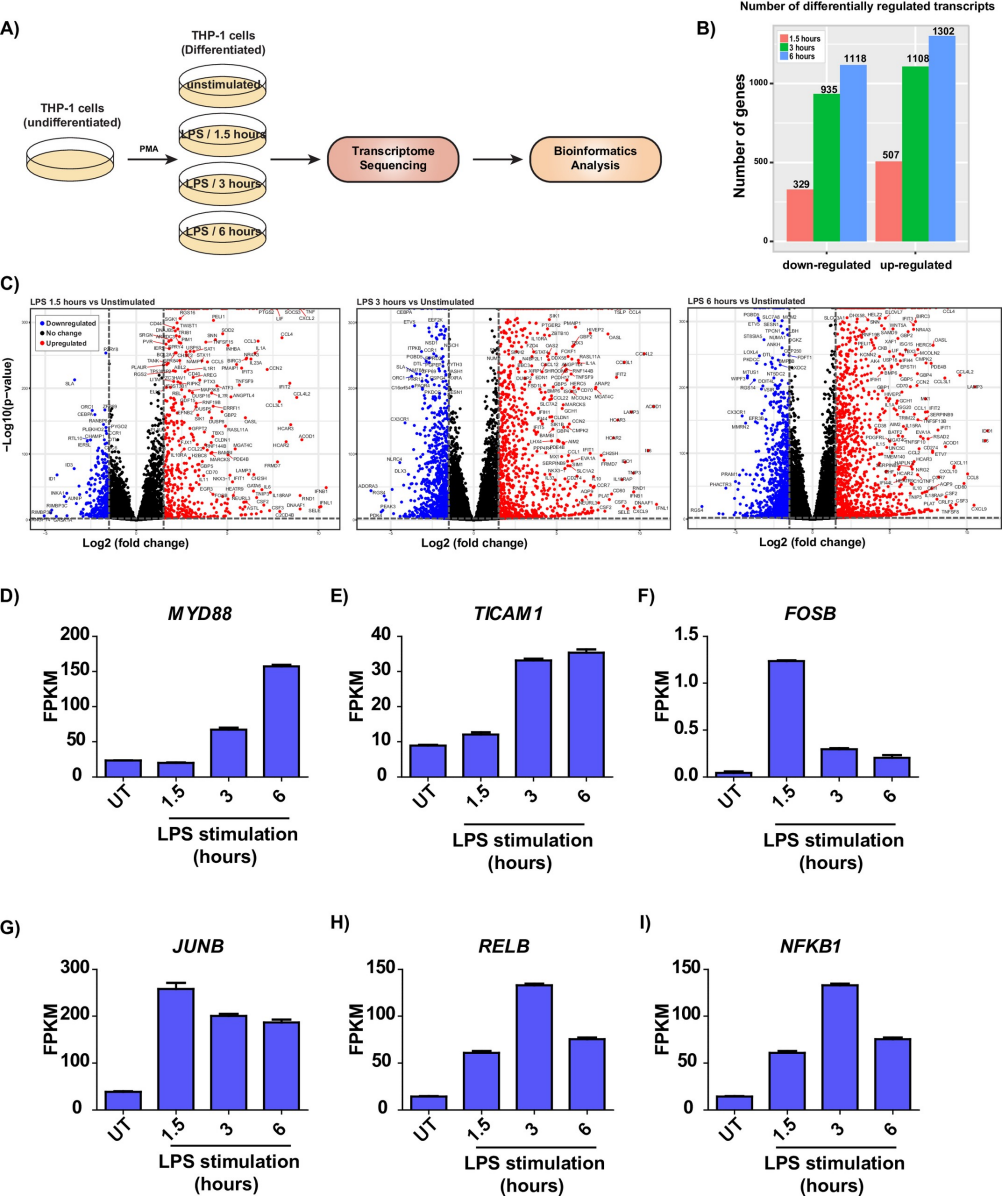
Temporal RNA-seq analysis of LPS-treated THP-1 macrophages. **(A)** Workflow employed for RNA-Seq. **(B)** Statistics of differentially regulated transcripts in response to LPS stimulation **(C)** Volcano plots depicting differentially expressed genes in response to temporal LPS stimulation. The points colored red, blue, and black represent upregulated, downregulated, and unchanged transcripts, respectively. Temporal expression of TLR-induced transcripts including **(D)** *MYD88* **(E)** *TICAM1* **(F)** *FOSB* **(G)** *JUNB* **(H)** *RELB* **(I)** *NFKB1*, in THP-1 macrophages in response to LPS stimulation.

To identify novel transcriptome dynamics induced by TLR4 activation, we carried out RNA-Seq analysis of PMA-differentiated human THP-1 cells temporally stimulated with the TLR4 ligand LPS for 1.5 hours, 3 hours, and 6 hours (**Fig 1A, S1 Table**). Correlation analysis of the transcriptome data indicated that replicate experiments were highly correlated (**S1 Fig**). Bioinformatics analysis of the transcriptomic data led to the identification of several differentially expressed genes (DEGs) (log_2_(fold-change) cutoff > = 1.5 OR < = -1.5; p-value cutoff < = 0.01) in LPS-stimulated macrophages (**Fig 1B and 1C**). Our analysis resulted in the identification of well-known markers of TLR4 activation and novel transcripts identified to be differentially expressed for the first in response to TLR4. Transcripts well-known to be induced by TLR4 activation, including *MYD88*, *TICAM1*, *FOSB*, *JUNB*, *RELB*, and *NFKB1* (**Fig 1D–1I**), amongst several others, were identified in the transcriptome data.

### Analysis of LPS-stimulated THP-1 macrophage transcriptome identifies known and potential markers of TLR-induced interferon response

**Fig 2:**
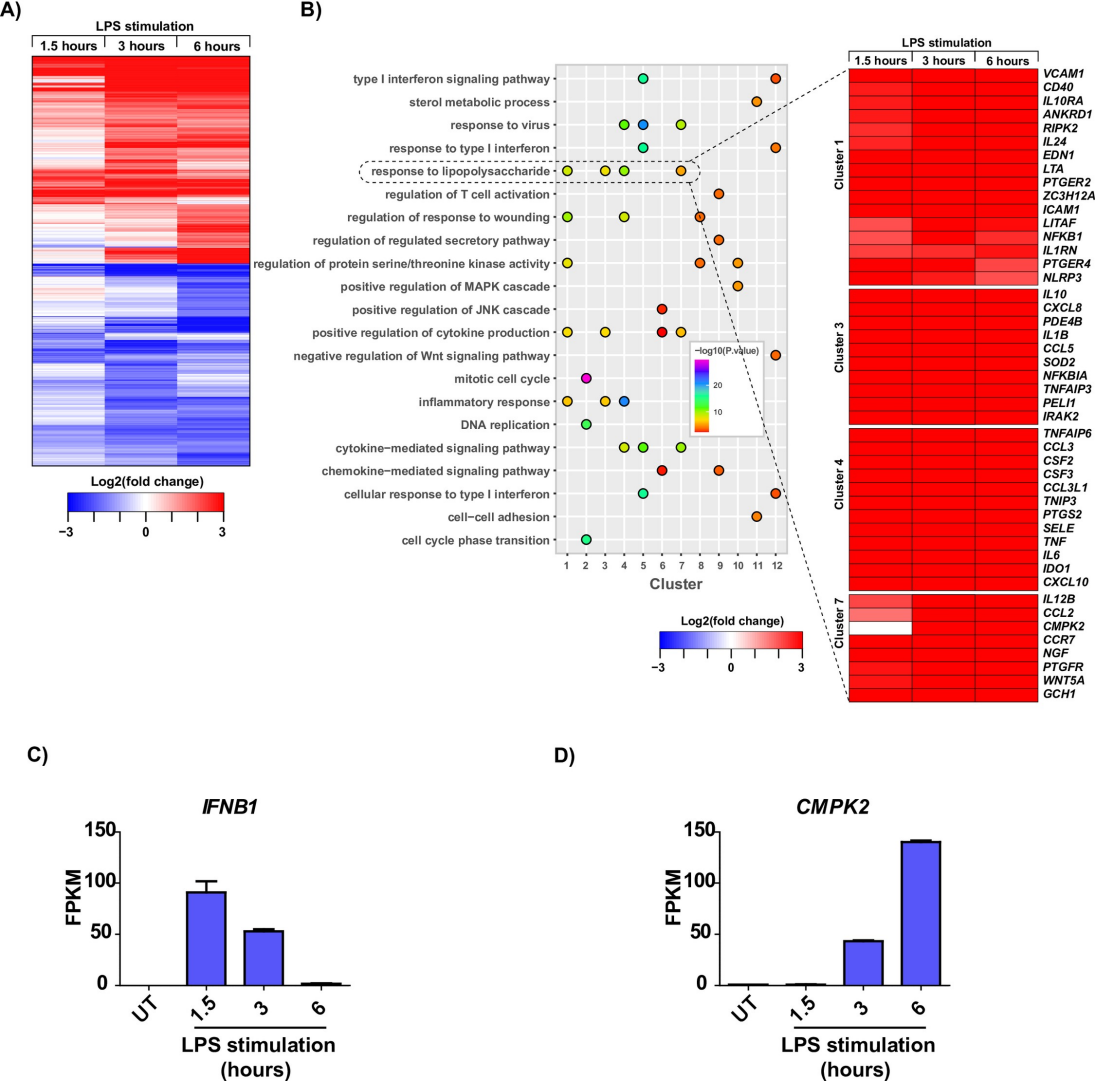
Analysis of RNA-seq data from LPS stimulated macrophages reveals dysregulation of multiple signaling pathways. **(A)** Heatstrip showing DEGs in response to temporal LPS stimulation **(B)** Gene set enrichment analysis revealed the enrichment of several pathways in response to LPS stimulation. Dotted lines highlight the dysregulation of gene clusters 1, 3, 4, and 7 belonging to the ‘response to lipopolysaccharide’ process. Temporal change of **(C)** *IFNB1* and **(D)** *CMPK2* transcripts in THP-1 macrophages in response to LPS stimulation.

A detailed observation of DEG expression dynamic patterns across the studied timepoints indicated clusters of common as well as timepoint-specific transcriptome changes (**Fig 2A**). Of these, 400 upregulated genes, and 164 downregulated common across all the timepoints. Analysis of these clusters revealed little overlap between gene clusters (**S2A and S2C Fig**). Significantly changing transcripts are represented in **S2B Fig**.

Next, we carried out a comparison of DEGs in response to LPS with gene sets encompassing Interferon responsive genes, response to interferon-alpha, beta, and gamma, transcription factors, inflammasome markers, cytokines, and chemokines. We observed a progressive increase in the expression levels of genes associated with all classes across time (**S2D Fig**). Several interferon responsive genes such as *IFIT*s (Interferon Induced proteins with Tetratricopeptide repeats), *ISG*s (interferon-stimulated genes), *IFNAR2* (interferon alpha and beta receptor subunit 2), *IFNB1* (interferon beta 1), *IFNGR2* (interferon gamma receptor 2), and *OASL* (2’-5’-oligoadenylate synthetase like) were found to be upregulated across all time points studied in response to LPS. Additionally, we also observed several cytokines and chemokines to be overexpressed in response to LPS, including previous examples known to be impacted by TLR4 stimulation such as *IL1A*, *IL1B*, *IL6*, *CXCL8 (IL8)*, *IL10*, *TNF*, amongst others. Transcription factors well-known in TLR signaling such as *FOSB*, *FOSL1*, *JUNB*, *NFKB1*, *NFKB2*, *REL*, and *RELB* were also found to be upregulated in response to LPS. NLRP3 inflammasome markers including *AIM2*, *CASP5*, and *NLRP3* were found to be upregulated while *NLRC4* and *NLRP1* were found to be downregulated in response to LPS, as reported in previous reports [39].

Gene-set enrichment analysis (GSEA) of LPS-induced DEGs led to the identification of enriched pathways and processes impacted by TLR activation (**Fig 2B**). Among the various pathways that were affected by LPS, the prominent ones included type I IFN signaling response, response to liposaccharide, and regulation of protein serine/threonine kinase activity. A thorough examination of the individual genes corresponding to the ‘response to lipopolysaccharide’ biological process led to the identification of cytidine/uridine monophosphate kinase 2 (*CMPK2*) as a potential TLR4-inducible gene. While *IFNB1* showed a temporal decrease in transcript levels, *CMPK2* showed a temporal increase in response to LPS stimulation (**Fig 2C and 2D**). Further examination into published literature on the role of CMPK2 in macrophage TLR signaling indicated that CMPK2 was a relatively less characterized but highly inducible gene. Taken together, transcriptome analysis of LPS stimulated THP-1 macrophages revealed upregulation of several known and novel genes pertaining to the TLR and Interferon signaling pathways, including *CMPK2*, the role of which in interferon signaling is less known. Therefore, we proceeded to characterize the regulation of CMPK2 expression downstream of TLR signaling in macrophages.

### CMPK2 is induced by LPS and Poly (I:C)

Earlier studies have indicated that CMPK2 is notably induced in response to LPS, Poly (I:C), as well as certain viruses, including HIV (human immunodeficiency virus) [40], HEV (hepatitis E virus) [41], PRRSV (porcine reproductive and respiratory syndrome virus) [42], and SVCV (spring viremia of carp virus) [43] in various cell types. However, the vast majority of these studies were performed in the context of epithelial cells or cells derived from other species, and very little is known of its expression dynamics in human macrophages. We assessed the CMPK2 regulatory mechanisms in THP-1 cells by examining the mRNA expression of *CMPK2* in response to TLR ligands: Pam3CSK4 (TLR1/2), FSL1 (TLR2/6), Poly (I:C) (TLR3), LPS K12 (TLR4), flagellin (TLR5), R837 (TLR7), R848 (TLR7/8), CL075 (TLR8), and CpG2006 (TLR9) using quantitative RT- PCR analysis.

**Fig 3:**
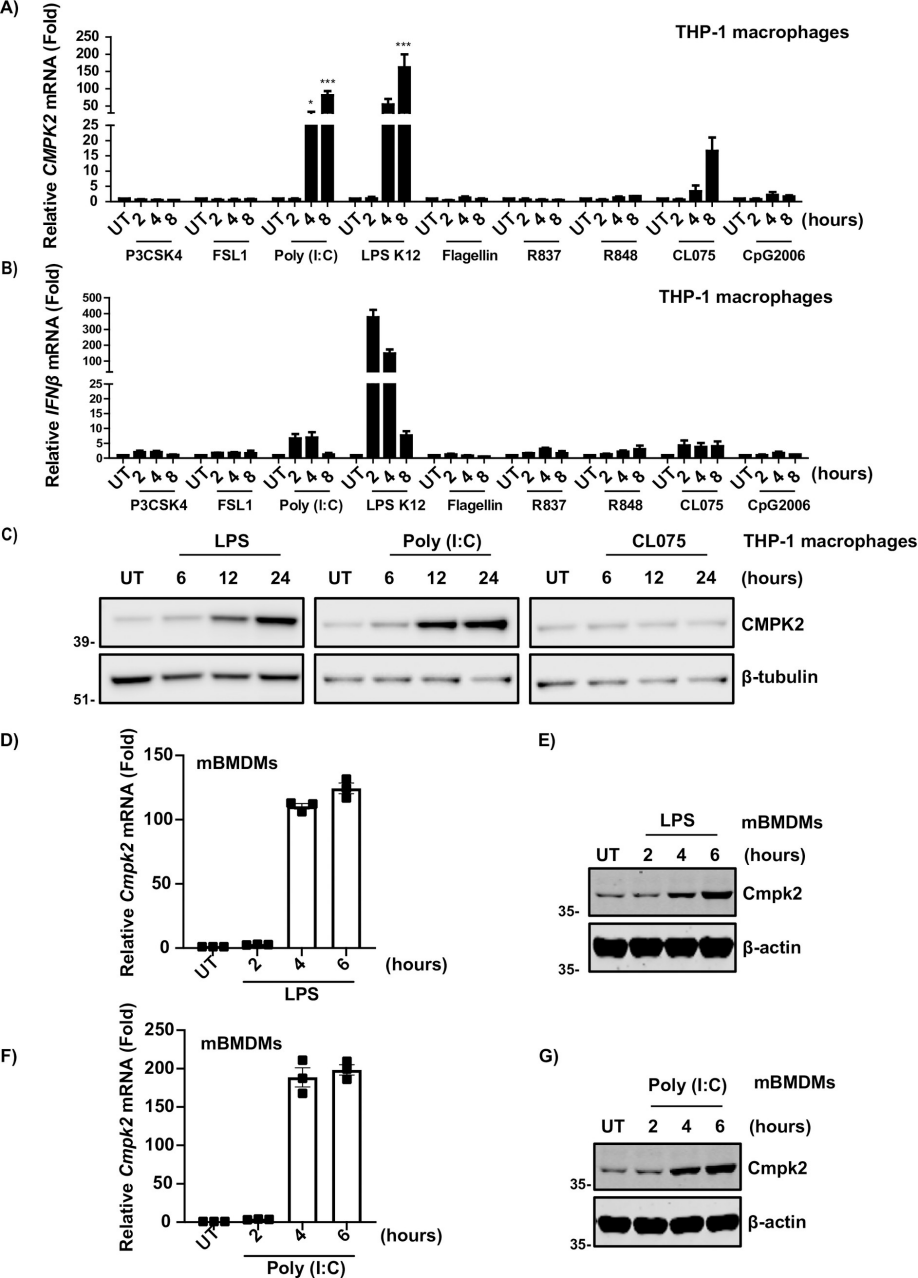
The expression pattern of CMPK2 is LPS and Poly (I:C) specific in THP-1 and mBMDMs. The mRNA expression of (**A**) *CMPK2* and (**B**) *IFNβ* in THP-1 cells standardized by 18s upon different TLR ligands: P3CSK4, FSL1, Poly (I:C), LPS K12, Flagellin, R837, R848, CL075, and CpG2006. The values are represented as the mean ± S.E. from three independent samples. (**C**) Western blot analysis of CMPK2 protein expression in THP-1 cells treated with LPS (200ng/ml), Poly (I:C) (10 μg/ml), and CLO75 (5 μg/ml) for the indicated time points. β-tubulin was used as the loading control. Quantitative real-time PCR of *CMPK2* gene expression in BMDMs before and after (**D**) LPS and (**F**) Poly (I:C) challenge (n = 3 biological replicates pooled). Immunoblot analysis of CMPK2 in BMDMs after treatment with (**E**) LPS and (**G**) (Poly (I:C). Detection of β-actin was used as a loading control. One-way analysis of variance (ANOVA) with Dunnett post analysis was employed for the statistical analysis. (*p, 0.05, ***p, 0.001).

As shown in **Fig 3A**, the transcript levels of *CMPK2* were upregulated as expected by LPS. Furthermore, we observed increased expression in response to Poly (I:C) and to a lesser extent with CL075 in macrophage‐like differentiated THP-1 cells. All three stimuli showed an increasing trend from 4 hours of stimulation and reached peak levels at the 8-hour time point. Of note, treatment with LPS resulted in a higher *CMPK2* mRNA level than Poly (I:C). The increasing trend of *CMPK2* mRNA observed in qPCR was similar to the levels of upregulation of the *CMPK2* gene in RNA seq data, thereby validating our findings from RNA-seq analysis. In addition, *CMPK2* upregulation was accompanied by the upregulation of *IFNβ* (**Fig 3B**). We further validated the expression of CMPK2 at the protein level by Western blotting. In agreement with the real-time PCR data, CMPK2 expression was induced by LPS and poly (I:C) in a time-dependent manner (both reached the peak at 24 hours); however, no significant changes were observed in response to CL075 stimulation (**Fig 3C**). The discrepancy of CMPK2 mRNA and protein level upon CL075 stimulation could be explained since several factors cannot be detected or predicted at the mRNA level such as rate of translation, protein degradation, post-translation modification, and proteolytic cleavages [44–47]. We also studied the expression of *CMPK1* in THP-1 macrophages, a gene similar in function to *CMPK2*, in response to various TLR ligands. There were no significant changes either in *CMPK1* transcript levels or in the protein expression (**S3A and S3B Fig**).

We further investigated the induction of *CMPK2* in wild-type murine bone marrow-derived macrophages (mBMDMs). Corroborating with previous studies [48] and our findings in THP-1 macrophages, a significant increase in the CMPK2 levels both at transcript and protein levels was observed upon LPS and Poly (I:C) treatment in mBMDMs (**Fig 3D–3G**). A noteworthy observation was that CMPK2 was induced at an earlier timepoint in mBMDMs than in THP-1 macrophages. Taken together, our results demonstrate that the expression of CMPK2 is specifically induced in response to activation of TLR3 and TLR4 signaling in both THP-1-derived macrophages and BMDMs.

### CMPK2 is localized in both the cytoplasm and the mitochondria

Previous studies investigating CMPK2 localization showed differences between species and cell types. In fish, CMPK2 was expressed in the cytoplasm of Fathead minnow (FHM) cells [43]. In mice, CMPK2 was detected both in the cytoplasm and mitochondria, whereas CMPK2 was found to be localized in the mitochondria of HeLa cells [48–50]. Since localization of CMPK2 has been shown to vary, we next evaluated the subcellular localization of CMPK2 in THP-1 cells by both confocal microscopy and subcellular fractionation methods.

**Fig 4:**
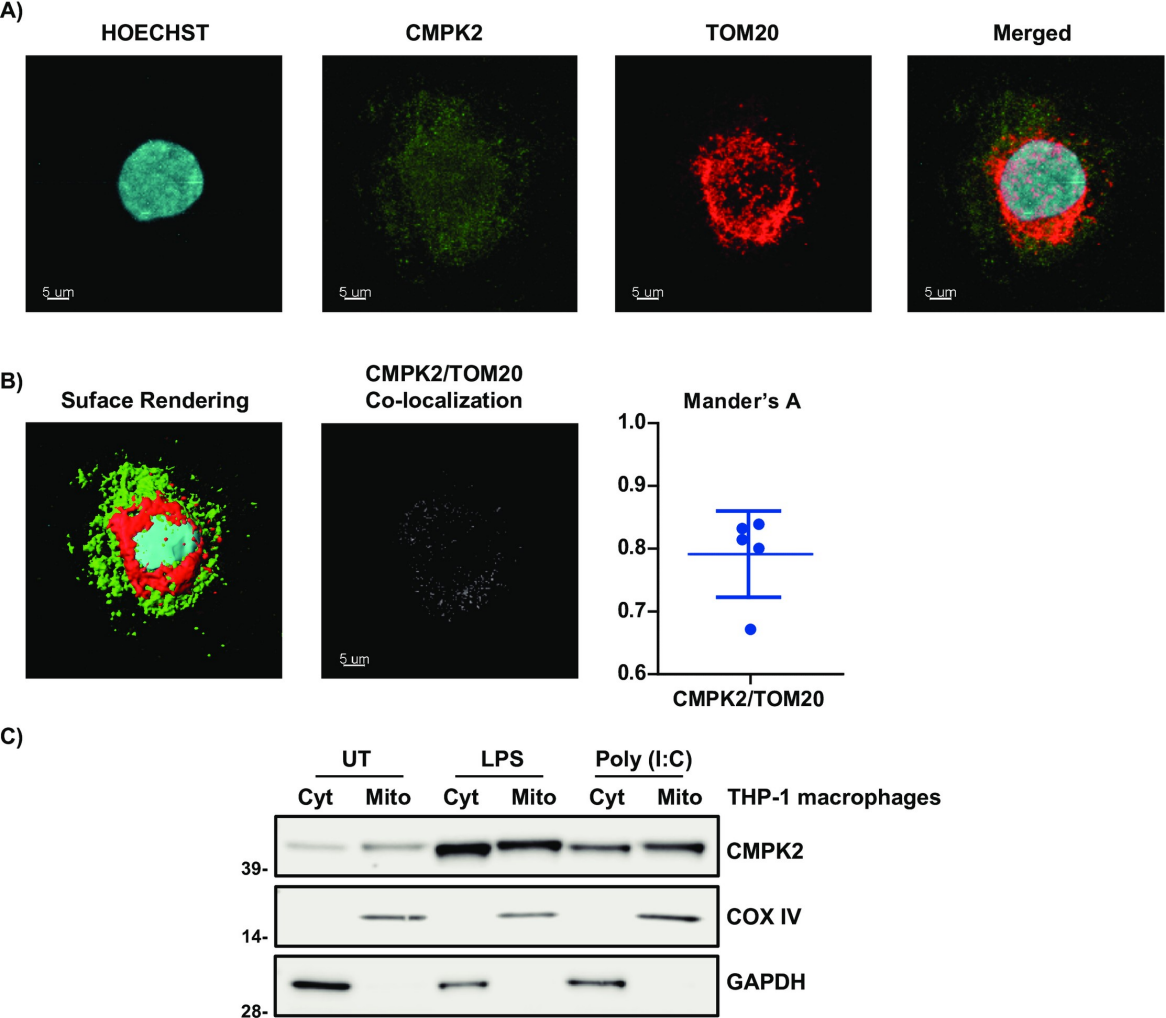
CMPK2 is predominantly localized in the cytoplasm *with* partial mitochondrial overlap. (**A**) THP-1 cells were subjected to confocal microscopy using anti-CMPK2, anti-TOM20 antibodies. Cell nuclei were stained with Hoechst. Scale bars: 5 μm. (**B**) Maximal projections and surface rendering of a z-stack is shown. Quantitative analysis of colocalization levels by Manders coefficient in THP-1 cells, analyzing the ratio between CMPK2 and TOM20. (C) The cytoplasmic (Cyt) and mitochondrial (Mito) fractions were prepared from THP-1 cells before and after 24 hours of LPS and Poly (I:C) stimulations and analyzed by Western blot for CMPK2. GAPDH and COX IV were used as markers for cytoplasmic proteins and mitochondrial inner membrane and intermembrane space proteins, respectively. Data are typical of three separate experiments.

As shown in **Fig 4A and 4B**, in unstimulated THP-1 cells, the majority of THP-1 cells showed distinct expression of CMPK2 in the cytoplasm but with partial overlap with mitochondrial marker TOM20, suggesting that CMPK2 is mainly distributed in the cytoplasm and partially in the mitochondria. The presence of CMPK2 in these organelles was further supported by immunoblotting results after subcellular fractionation (**Fig 4C**). Consistently, CMPK2 expression was observed both in cytosolic and mitochondrial fractions in untreated THP-1 cells. Moreover, when the THP-1 cells were stimulated with LPS and Poly (I:C) for 24 hours, expression of CMPK2 protein was further increased in both the cytoplasm and the mitochondrial fractions. Of note, the level of cytosolic CMPK2 expression (CMPK2/GAPDH) was generally greater than the level of mitochondrial CMPK2 expression (CMPK2/COXIV). Also, the levels of both cytosolic and mitochondrial CMPK2 expression induced by LPS were higher than Poly (I:C) (**S4A and S4B Fig and Fig 4C**). These data imply that CMPK2 is distributed in both cytoplasm and mitochondria but localized predominantly in the cytoplasm of THP-1 cells.

### Induction of CMPK2 by LPS and Poly (I:C) is type I interferon response-dependent

**Fig 5:**
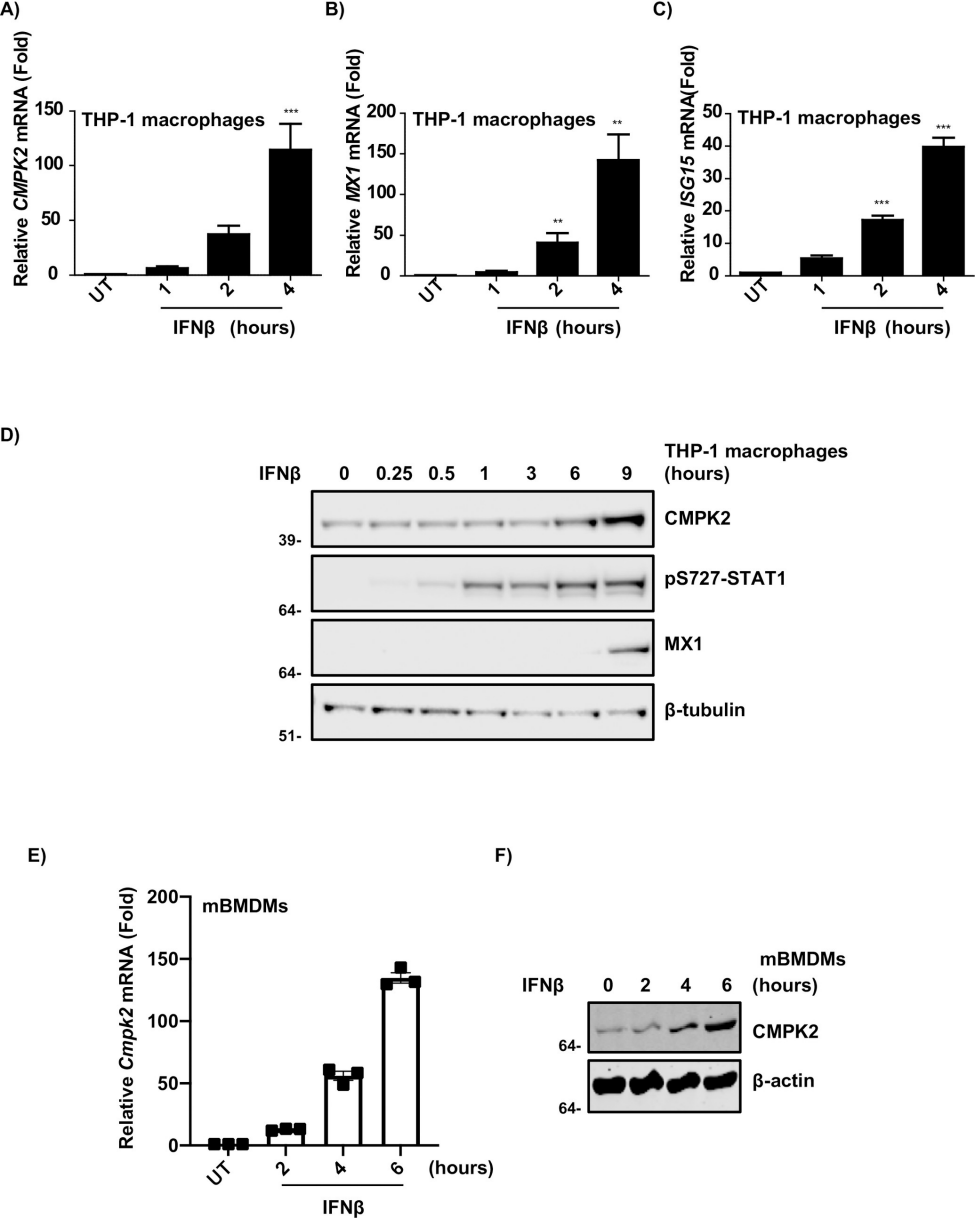
IFN-β treatment induces CMPK2 expression in THP-1 and BMDMs. Quantitative RT-PCR of (**A**) *CMPK2*, (**B**) *MX1*, and (**C**) *ISG15* in THP-1 cells stimulated with IFN-β (100 units/ml) for the indicated time. **(D)** Representative CMPK2, pStat1, and MX1 protein levels of THP-1 cells treated with IFN-β over a 0.25–9 hours time course. β-tubulin was used as a loading control. **(E)** Quantitative real-time PCR of *CMPK2* and (**F**) Western blots of CMPK2 in mBMDMs, 2–6 hours after stimulation with IFN-β (10ng/ml) (n = 3 biological replicates pooled). β-actin was used as a loading control. Error bars represent the mean ± SEM of three independent experiments, and all figures are representative of three independent experiments. One-way analysis of variance (ANOVA), followed by Dunnett’s post hoc analysis, was employed for the statistical analysis. (**p, 0.01, ***p, 0.001).

The activation of macrophages by LPS and Poly (I:C) initiates the host innate immune response and induces type I IFNs and pro-inflammatory cytokines. Type I IFNs bind to the heterodimeric IFN receptors (IFNARs) and results in the activation of tyrosine kinase 2 (TYK2) and Janus Kinase 1 (JAK1), which in turn, phosphorylate signal transducer and activator of transcription 1 and 2 (STAT1 and STAT2) [8]. This transduction cascade leads to the induction of interferon-stimulated genes (ISGs). CMPK2 is a known ISG and was highly expressed upon LPS and Poly (I:C) challenges in our expression pattern study. Hence, we first confirmed the ability of IFN-β (Type I IFN) to induce CMPK2 and other ISG expressions in THP-1 cells by RT-qPCR and Western blot analysis. THP-1 cells were treated with IFN-β for various durations between 1–4 hours, and as expected, mRNA levels of *CMPK2* along with two classical ISGs- *MX1* and *ISG15* were significantly increased at 4 hours with IFN-β treatment (**Fig 5A–5C**). Treatment of THP-1 cells with IFN-β markedly upregulated MX1 protein level at 9 hours (**Fig 5D**). Both CMPK2 and phospho-STAT1 (S727) showed increasing expression trends with a maximal peak observed at 9 hours (**Figs 5D, S5A and S5B**). Furthermore, treatment of IFN-β induced upregulation of transcriptional and translational expression of CMPK2 in mBMDMs (**Fig 5E and 5F**). Consistent with previous findings, this data confirms that CMPK2 is an IFN-inducible gene in both humans and mice.

### CMPK2 induction is IRF-3 and interferon-α/β receptor (IFNAR)-dependent

**Fig 6:**
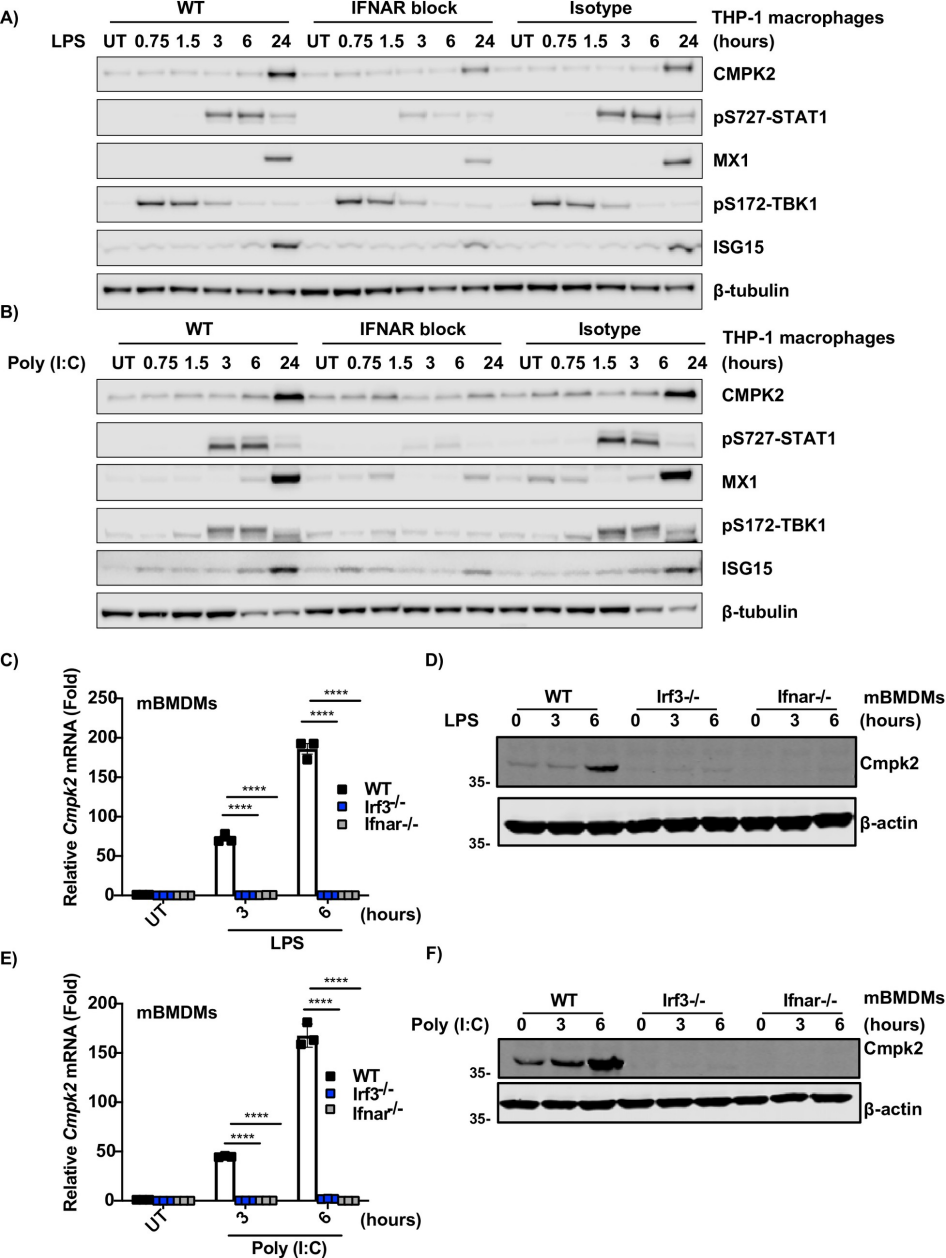
IFNAR and IRF3 are involved in the regulation of LPS and Poly (I:C)-mediated CMPK2 mRNA and protein expression. THP-1 cells were treated with control mAbs (MOPC-173) or IFNAR chain 2 mAbs for 30 minutes, then stimulated with (**A**) LPS and (**B**) Poly (I:C). Lysates were used for Western blotting to detect CMPK2, p-Stat1, MX1, p-TBK1, and ISG15. β-tubulin was used as an equal loading control. (**C**) LPS and (**E**) Poly (I:C) treated wild-type, *Irf3*^*−/−*^, and *Ifnar1*^*−/−*^ BMDMs mRNA expression of *CMPK2* was measured by quantitative PCR. Immunoblot analysis of CMPK2 in lysates of wild-type, *Irf3*^*−/−*^, and *Ifnar1*^*−/−*^ BMDMs after treatment with (**D**) LPS and (**F**) Poly (I:C). Error bars represent means ± SEM from three independent samples. All results were from three independent experiments.

To determine the role of IFN-β in the induction of CMPK2 expression by LPS and Poly (I:C) in THP-1 cells, we used a neutralizing antibody for Interferon-α/β receptor (IFNAR) blockade. As expected, the expression levels of CMPK2, phospho-STAT1 (S727), MX1, and ISG15 were significantly increased upon LPS and Poly (I:C) stimulation in both untreated and MMHAR-2 isotype treated THP-1 cells (**Fig 6A and 6B**). Phospho-TBK1 (S172), the activator of IRF3, showed an early increase and progressively decreased across time. On the other hand, IFNAR blockade remarkably reduced the expression of CMPK2, indicating that the expression of CMPK2 is driven by LPS and Poly (I:C)–type 1 Interferon mediated signaling axis (**Figs 6A, 6B, S6A and S6B**).

The key transcription factor that induces the production of IFNβ downstream of TLR3 and TLR4 is interferon regulatory factor 3 (IRF3) [51]. Hence, in a related set of experiments, we prepared BMDMs from WT, *Irf3*^−/−^ and *Ifnar1*^−/−^ mice and treated the cells with LPS and Poly (I:C) for 3 hours and 6 hours. Interestingly, CMPK2 mRNA and protein expression was abolished entirely in *Irf3*^−/−^ and *Ifnar1*^−/−^ macrophages (**Fig 6C–6F**). Therefore, our findings demonstrate a critical requirement for IRF-3- type I IFN in the induction of CMPK2 expression in LPS and Poly (I:C) stimulated THP-1 cells and BMDMs.

We also determined if RIG-1 and STING pathways had a role in inducing CMPK2 expression since these can robustly induce IRF3 and IFNβ pathways (**S7A–S7D Fig**). THP-1 macrophages treated with the STING ligand cGAMP showed increased *CMPK2* and *IFNβ* mRNA levels. Further, mBMDMs infected with Sendai virus (RIG-1 pathway) [52, 53] and HSV-1 (cGAS-STING pathway) [54] and mBMDMs treated with STING ligand diABZI showed increased *CMPK2* and *IFNβ* mRNA levels. These findings demonstrate that CMPK2 expression can also be triggered by RIG-1 and STING-induced Interferon pathways.

## Discussion

Initiation of innate immune response to various infectious pathogens involves the association of intricate networks of signal transduction pathways, leading to the transcription of specific sets of genes. LPS is a potent regulator of TLR4 signaling in macrophages, however, only a limited number of studies have explored time-dependent LPS-mediated gene regulation in THP-1 macrophage-like cells [55, 56]. These studies focused on the identification of promoter-enhancer interactions or identifying molecular mechanisms of parasite infection. In the present study, we aimed to identify interferon-stimulated genes, most importantly, the relatively less characterized interferon-stimulated genes concerning the TLR pathway. Notably, we identified CMPK2 as a differentially expressed gene in LPS treated THP-1 cells. CMPK2, also called UMP-CMP kinase 2, is an enzyme of pyrimidine metabolism that phosphorylates pyrimidine phosphates, including dUMP, dCMP, CMP, UMP, dUTP, and dCTP. We found that TLR3 and TLR4 activation induce drastic up-regulation of CMPK2 expression at both mRNA and protein levels in THP-1 cells and BMDMs. Subsequent verification and characterization demonstrated that CMPK2 is mainly localized in the cytoplasm and partially in the mitochondria in THP-1 cells. We also confirmed that CMPK2 is IFN-inducible gene, and found that its expression is mediated through TLR3/TLR4-IRF3-IFNβ-IFNAR signaling. The induction kinetics presented in this study suggests that CMPK2 may be induced through the LPS and Poly (I:C) mediated IRF3-type I IFN signaling pathway and is involved in the immune response against both bacterial and viral infections.

According to the previous exploration, CMPK2 was upregulated in response to the LPS in macrophages [48] and various types of viruses [40, 43, 57]. Our RNA seq analysis in THP-1 and expression study in THP-1 cells and BMDMs showed LPS and Poly (I:C) dependent CMPK2 expression. Hence, our observations are consistent with the previous findings. Information on protein subcellular localization reveals valuable insights into its regulation and function. Since the discovery of CMPK2, several studies have examined its localization in various species and cell types. An early study demonstrated that CMPK2 is localized in mitochondria of HeLa cells [50], while another study using Fathead minnow (FHM) cells suggests it is localized to the cytoplasm [43]. In addition, the current study unequivocally demonstrates that it is localized in both subcellular locations in THP-1 cells. A previous study showed that CMPK2 mRNA was readily detectable in monocyte/macrophage differentiating cell lines, such as THP-1 cells, but not in HeLa cells, a human cervical cancer cell [49]. This suggests the possibility that CMPK2 expression is species-specific. While higher vertebrates share a high level of sequence similarities, early vertebrates may have low levels of sequence similarities [49].

Notably, CMPK1, cytidine/uridine monophosphate kinase 1, an enzyme that has same principal role as CMPK2 in nucleotide synthesis, also resides in the cytosol. However, we found that CMPK1 mRNA levels were unchanged with all TLR ligands, suggesting that only CMPK2 has a specialized cellular function in the context of the innate immune response. Some genes have multiple subcellular localization sites and following specialized immune functions. For instance, mitochondrial anti-viral signaling proteins (MAVS) are localized in the outer membrane of the mitochondria, peroxisomes, and endoplasmic reticulum (ER), and each location has different anti-viral signaling functions. Mitochondrial MAVS signals the induction of IFN-β, type I IFN, while peroxisomal MAVS signals the induction of the type III IFNs [58]. It is unclear whether CMPK2 has any function in the cytosol, but we infer CMPK2 is bifunctional or cytosolic, and mitochondrial CMPK2 is coregulated through as-yet-unknown mechanism(s), which may contribute to the immune responses.

Due to mitochondrial targeting sequence in the N-terminal of CMPK2, several studies have focused on the mitochondrial function of CMPK2. Recently, Zhong *et al*. demonstrated that mitochondrial CMPK2 is involved in LPS-induced mitochondrial DNA (mtDNA) synthesis in BMDMs by supplying deoxyribonucleotides [48]. The newly synthesized DNA is oxidized by reactive oxygen species (ROS), then released into the cytosol and activates NLRP3 inflammasome [48]. In addition to activating the inflammasome, mtDNA can also trigger an interferon response [59]. Here, we hypothesize that this oxidized mtDNA in the cytosol may also activate cytosolic sensor -cyclic GMP‐AMP synthase (cGAS)- stimulator of interferon genes (STING), leading to the phosphorylation of transcription factor IRF3 and induction of type I IFNs [60]. Then possibly, the secreted type I IFNs can diffuse and signal to cells expressing IFNAR to amplify the response via expression of CMPK2.

Interferon regulatory factors (IRFs), such as IRF1, IRF3, IRF5, and IRF7 are the transcriptional regulators of type I IFNs and IFN-inducible genes in TLR signaling [61, 62]. One of the notable findings in our study was that CMPK2 expression is IRF3 dependent. This was based on our observations that TBK1, the upstream activator of IRF3, was activated early, and *Irf3*^−/−^ BMDMs abolished CMPK2 expression. In addition, TLR agonists such as LPS and Poly (I:C) are identified as inducers of type I IFN gene expression through the interferon regulatory factor 3 (IRF3) transcription factor [63]. This is in striking contrast to a previous study showing IRF1-dependent transcription of CMPK2 [48]. IRF family members share sequence homology in their DNA binding domains [64]. Like IRF3, IRF1 binds to DNA elements such as interferon regulatory element (IRE) sequences and activates the transcription of antiviral ISGs [65]. Collectively, this suggests that there are two independent or complementary regulatory pathways controlling CMPK2 gene expression.

Type I IFNs induce IFN-stimulated gene (ISG) expression to establish a cellular anti-viral/anti-bacterial state. Viperin (*RSAD2*) is a better-characterized ISG that displays anti-viral activities. In a previous study, similar to the CMPK2 expression trend shown in our study, Viperin expression was completely abolished in *Ifnar1*^*-/-*^, and *Irf3*^*-/-*^ mice. In fact, CMPK2 is adjacent to the Viperin gene in vertebrates and known to be transcribed along with Viperin during IFN stimulation [66–68]. This suggests that CMPK2 may, like Viperin, have anti-bacterial and anti-viral functions. Type I IFNs have an important role in both anti-bacterial and anti-viral properties. However, excessive production of type I IFNs can lead to the pathogenesis of autoimmune disorders, such as dermatomyositis (DM), lupus nephritis (LN), cutaneous lupus erythematosus (CLE), and systemic lupus erythematosus (SLE) [69–72]. Interestingly, CMPK2 gene expression was shown to be highly upregulated in childhood type DM and SLE patients [73, 74]. In addition, CMPK2 was also shown to potentially contribute to premature atherosclerosis in SLE [75]. Although further investigation is required, this may indicate that CMPK2 may serve as a reasonable therapeutic target against different autoimmune disorders. The anti-viral and anti-bacterial effect of CMPK2 has been revealed recently [13, 40, 43, 48, 76]; however, underlying mechanisms remain largely unclear. While the antiviral role of CMPK2 is well-mapped, the anti-bacterial role has not been explored well with several anecdotal evidences of *CMPK2* upregulation in bacteria-infected macrophages. For example, a previous transcriptomics study showed the upregulation of CMPK2 in *M*. *tuberculosis*-infected THP-1 macrophages compared to uninfected controls [77]. Further *Cmpk2* was also found to be upregulated in RAW 264.7 macrophages infected with *Brucella abortus* compared to uninfected controls [78]. Further, CMPK2 in fish was shown to be involved in host innate immunity and plays a protective role in antimicrobial responses during bacterial infections [76]. Therefore, further experimental evidence is required to confirm the potential anti-viral and anti-bacterial roles of CMPK2.

Collectively, our findings provide insight into the potential anti-viral and anti-bacterial roles of CMPK2. The information presented in this study could guide future studies on the role of CMPK2 in type -1 IFN dependent anti-viral and anti-bacterial response and may provide a novel potential therapeutic target to the treatment of various type I IFN related autoimmune disorders.

## Supporting information

S1 Fig Comparison of transcriptome profiles of replicates indicates high levels of correlation in **(A)** Unstimulated macrophages and those treated with LPS for **(B)** 1.5 hours**, (C)** 3 hours, and **(D**) 6 hours.
(TIF)

S2 Fig RNA-seq analysis identifies CMPK2 as a TLR4-induced gene. **(A)** Principal Component Analysis (PCA) plot depicting clusters of similarly expressed transcripts in THP-1 cells in response to LPS stimulation **(B)** Heatstrip depicting transcripts that were significantly changing in THP-1 macrophages across time in response to LPS stimulation. **(C)** DEG expression dynamic patterns across the studied timepoints indicates clusters of common as well as timepoint-specific transcriptome changes **(D)** Bar graph depicting a temporal increase of specific gene sets in response to LPS stimulation.
(TIF)

S3 Fig CMPK1 mRNA expression levels are constant upon TLR ligand stimulations. **(A)** Quantitative real-time PCR of CMPK1 gene expression in THP-1 cells treated with different TLR ligands for indicated time point. The samples were collected from three independent times. **(B)** Representative western blot showing CMPK2 expression in THP-1 cells after LPS, Poly (I:C), and CL075 challenge. β-tubulin was used as the loading control. Error bars represent the mean ± SEM of three independent experiments, and data were from three separate experiments.
(TIF)

S4 Fig CMPK2 is expressed in both cytoplasmic and mitochondrial fractions. Semi-quantification of the Western blot analysis from THP-1 fractionation as intensity ratios of (**A**) cytosolic CMPK2/GAPDH and (**B**) mitochondrial CMPK2/COXIV. Data are presented as mean ± SEM (n  =  3). Western blotting results were quantified with ImageJ.
(TIF)

S5 Fig IFNB stimulation leads to a significant increase in CMPK2 and p-STAT1 protein levels. (**A**) CMPK2 and (**B**) p-STAT1 expression values from IFNβ stimulated THP-1 cells Western blot images were quantified by ImageJ and were normalized to β-tubulin expression. Bars represent the mean ± SEM of three independent experiments. Statistical significance was determined by ANOVA, followed by Dunnett’s post-analysis (**p, 0.01, ***p, 0.001).
(TIF)

S6 Fig Blocking type I interferon signaling reduces CMPK2 expression. THP-1 cells preincubated with control mAbs (MOPC-173) or IFNAR chain 2 mAbs were stimulated with (**A**) LPS and (**B**) Poly (I:C). Following lysates were subjected to western blot analysis. Western blotting results were quantified with ImageJ and presented as the CMPK2/ β-tubulin ratio. Bars represent the mean ± SEM of three independent experiments.
(TIF)

S7 Fig Activation of cGAS-STING pathway and RIG-1 pathway increases interferon-induced CMPK2 expression. THP-1 cells were transfected with STING ligand, 2’3’-cGAMP (1 ug/ml) using Lipofectamine 3000 then assayed for (**A**) *CMPK2* and (**B**) *IFNβ* mRNA expression. Further, mBMDMs were infected with Sendai virus (RIG-1, 10 HA units/ml), Herpes Simplex Virus 1 (HSV1) (cGAS-STING pathway, MOI = 1) and treated with STING agonist–diABZI STING agonist-1 were assayed for (**C**) *CMPK2* and (**D**) *IFNβ* mRNA expression. Bars represent the mean ± SEM of three independent experiments.
(TIF)

S1 Table Summary of RNA sequencing (RNA seq) data of THP-1 macrophages treated with LPS for 1.5, 3, and 6 hours. (XLSX)

S1 Data (ZIP)

## Abbreviations

ATP: Adenosine triphosphate
BMDMs: bone marrow-derived macrophages
CMP: Cytidine monophosphate
CMPK2: Cytidine/uridine monophosphate kinase 2
CTP: Cytidine triphosphate
dCMP: Deoxycytidine monophosphate
DEG: Differentially expressed gene
dUMP: Deoxyuridine monophosphate
ENCODE: Encyclopedia of DNA Elements
GSEA: Gene-set enrichment analysis
IFNAR: Interferon-alpha/beta receptor
ISG: interferon-stimulated gene
LPS: Lipopolysaccharide
LPSK12: Lipopolysaccharide from Escherichia coli K12
NGS: Next-Generation Sequencing
PAMP: Pathogen-associated molecular patterns
PMA: Phorbol 12-myristate 13-acetate
Poly(I:C): Polyinosinic:polycytidylic acid
ROS: Reactive oxygen species
TLR: Toll-like receptor
UMP: Uridine monophosphate
UTP: Uridine triphosphate
