# Supplementary figures and images for: UMP-CMP kinase 2 gene expression in macrophages is dependent on the IRF3-IFNAR signaling axis
Source: PLoS One. 2021 Oct 27;16(10):e0258989. doi: 10.1371/journal.pone.0258989 (PMC8550426; doi:10.1371/journal.pone.0258989)

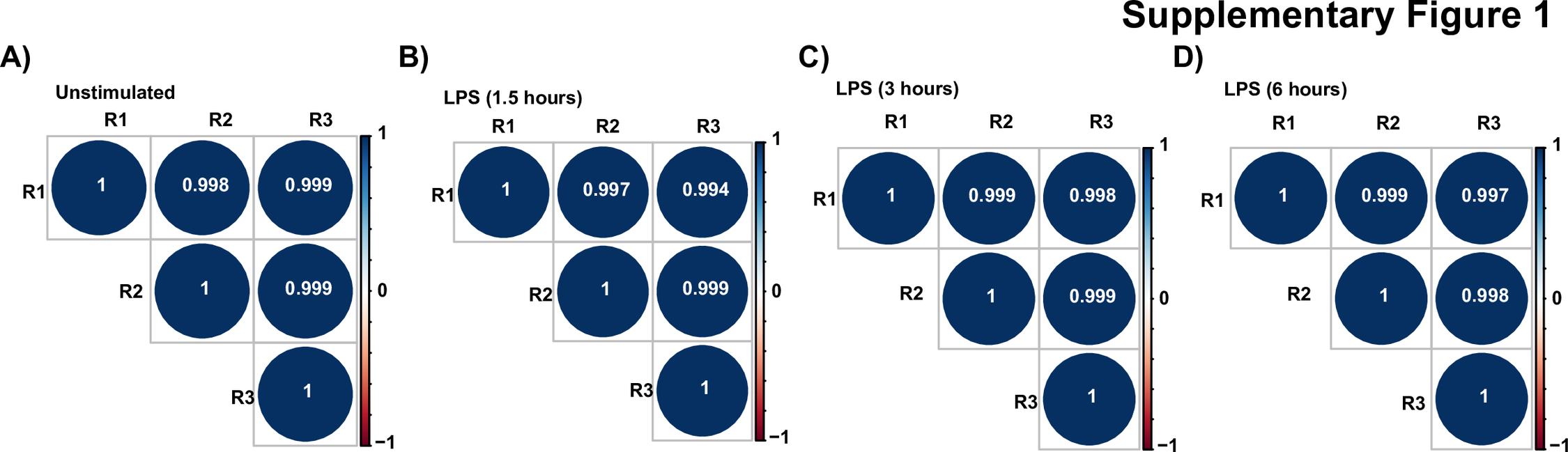

Supplement: S1 Fig — Comparison of transcriptome profiles of replicates indicates high levels of correlation in (A) Unstimulated macrophages and those treated with LPS for (B) 1.5 hours, (C) 3 hours, and (D) 6 hours. (TIF) [file pone.0258989.s001.tif]

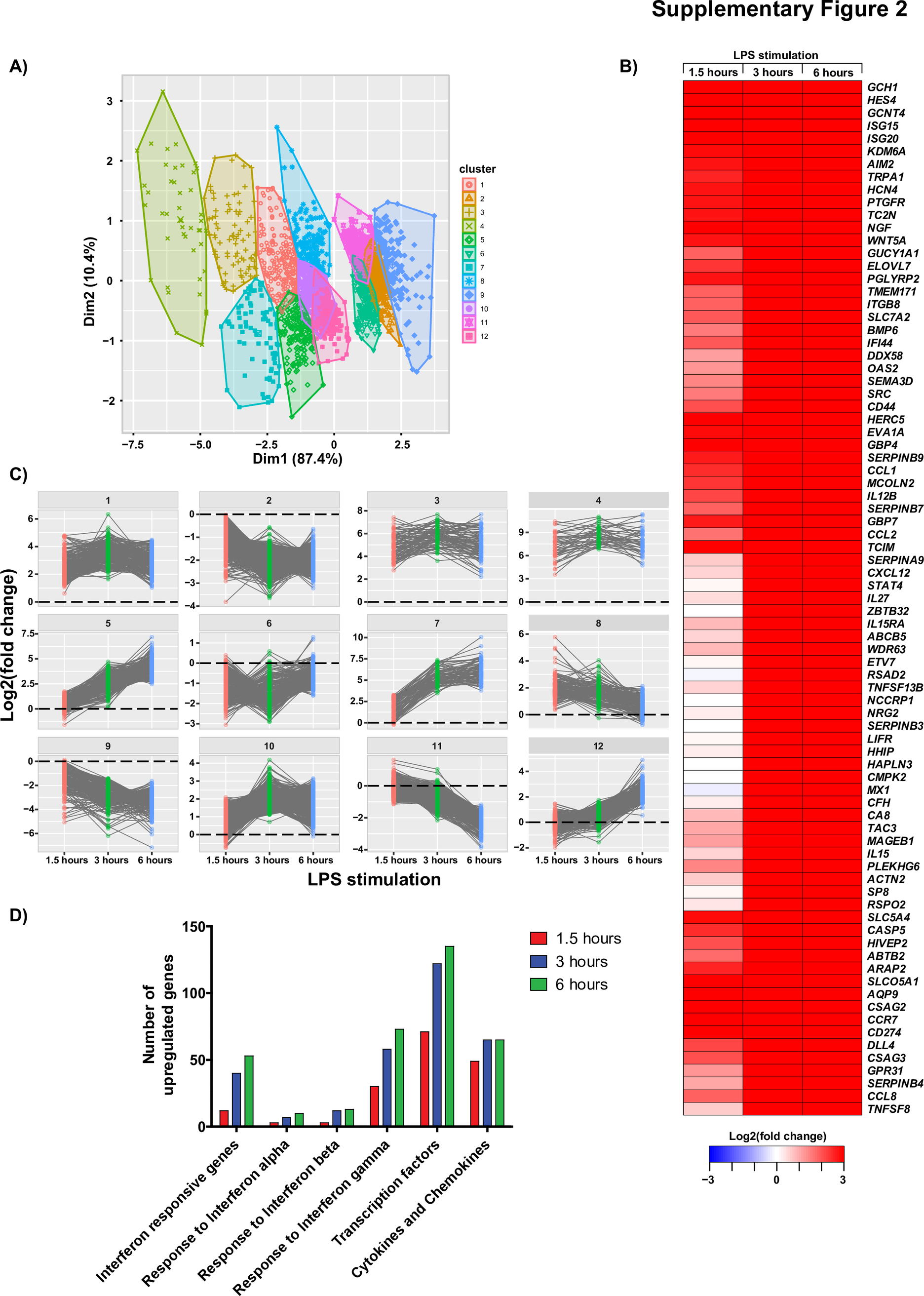

Supplement: S2 Fig — (A) Principal Component Analysis (PCA) plot depicting clusters of similarly expressed transcripts in THP-1 cells in response to LPS stimulation (B) Heatstrip depicting transcripts that were significantly changing in THP-1 macrophages across time in response to LPS stimulation. (C) DEG expression dynamic patterns across the studied timepoints indicates clusters of common as well as timepoint-specific transcriptome changes (D) Bar graph depicting a temporal increase of specific gene sets in response to LPS stimulation. (TIF) [file pone.0258989.s002.tif]

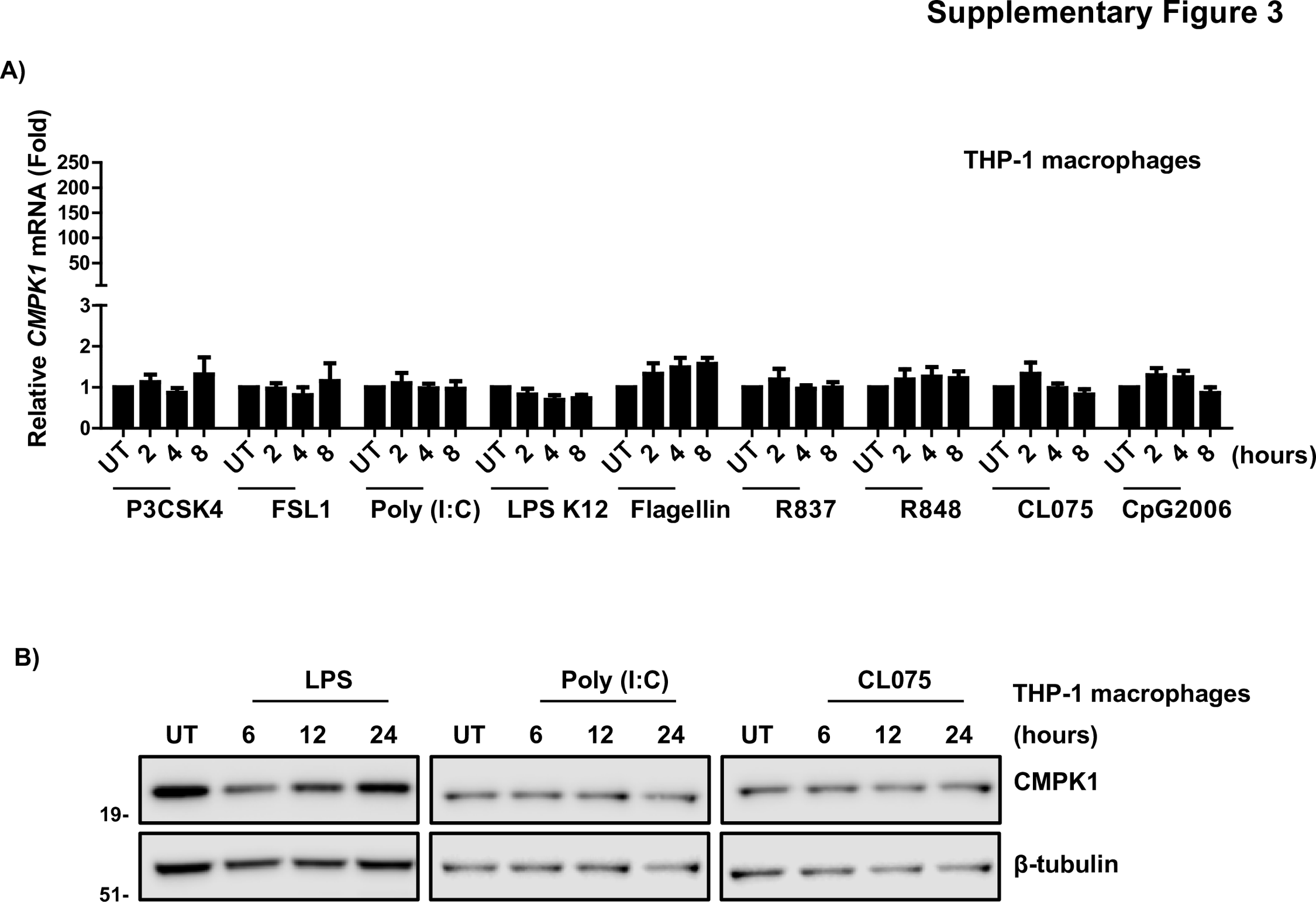

Supplement: S3 Fig — (A) Quantitative real-time PCR of CMPK1 gene expression in THP-1 cells treated with different TLR ligands for indicated time point. The samples were collected from three independent times. (B) Representative western blot showing CMPK2 expression in THP-1 cells after LPS, Poly (I:C), and CL075 challenge. β-tubulin was used as the loading control. Error bars represent the mean ± SEM of three independent experiments, and data were from three separate experiments. (TIF) [file pone.0258989.s003.tif]

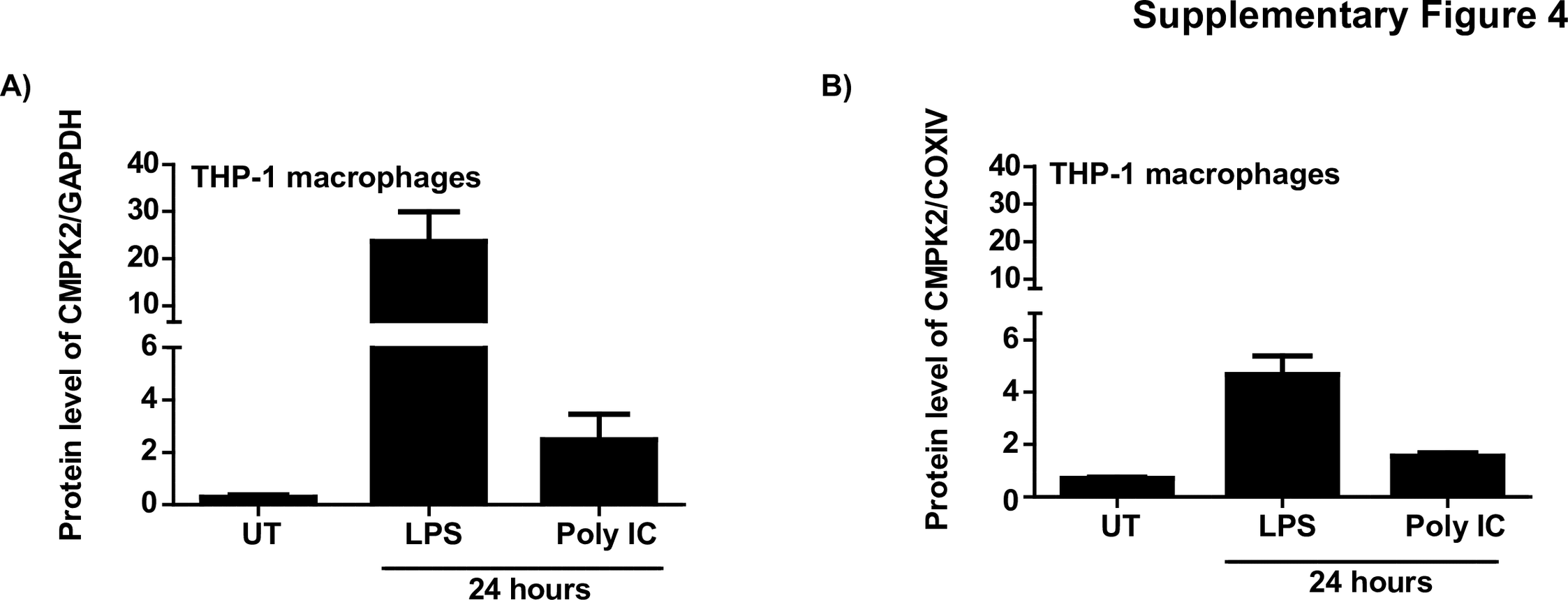

Supplement: S4 Fig — Semi-quantification of the Western blot analysis from THP-1 fractionation as intensity ratios of (A) cytosolic CMPK2/GAPDH and (B) mitochondrial CMPK2/COXIV. Data are presented as mean ± SEM (n = 3). Western blotting results were quantified with ImageJ. (TIF) [file pone.0258989.s004.tif]

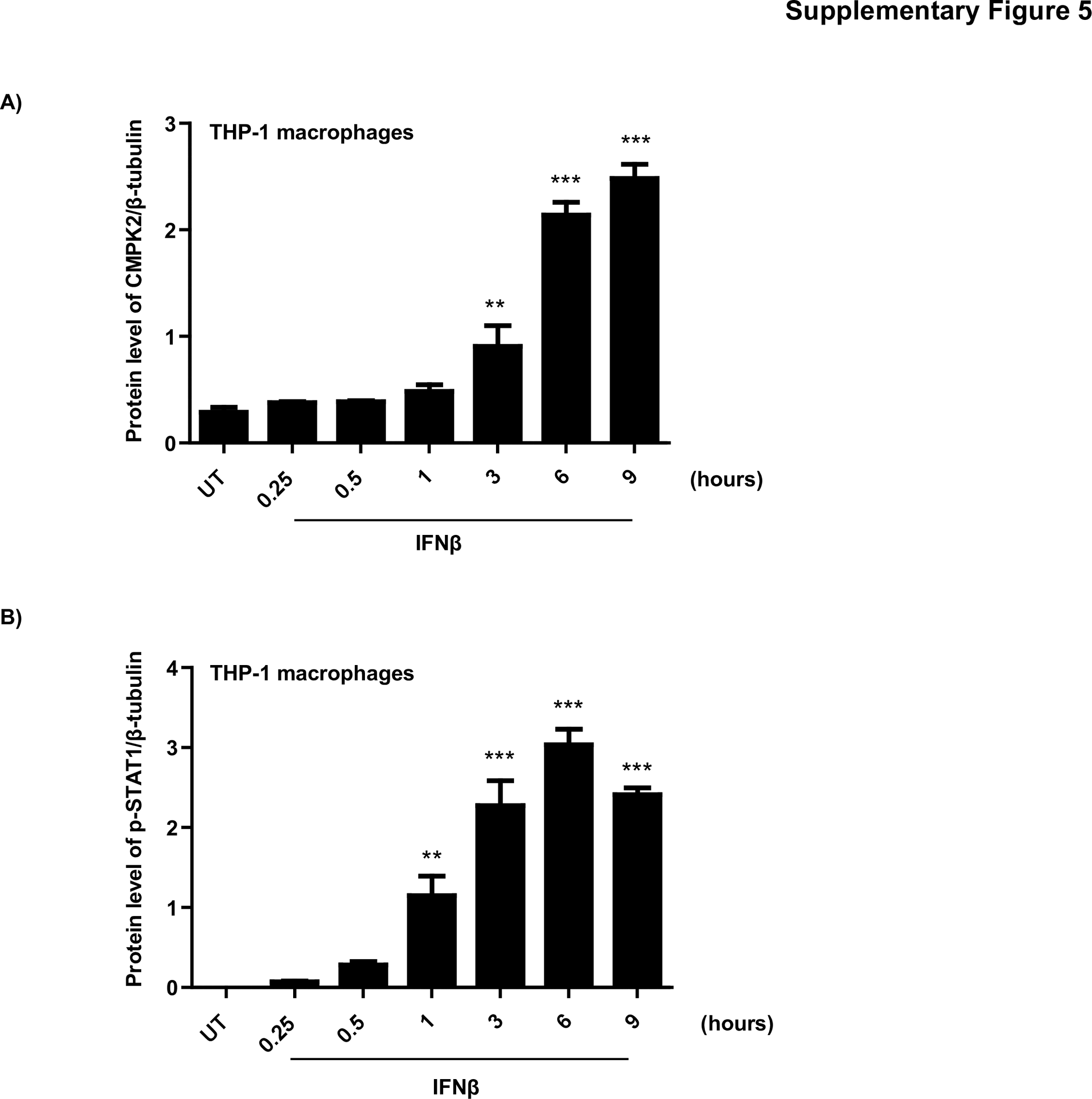

Supplement: S5 Fig — (A) CMPK2 and (B) p-STAT1 expression values from IFNβ stimulated THP-1 cells Western blot images were quantified by ImageJ and were normalized to β-tubulin expression. Bars represent the mean ± SEM of three independent experiments. Statistical significance was determined by ANOVA, followed by Dunnett’s post-analysis (**p, 0.01, ***p, 0.001). (TIF) [file pone.0258989.s005.tif]

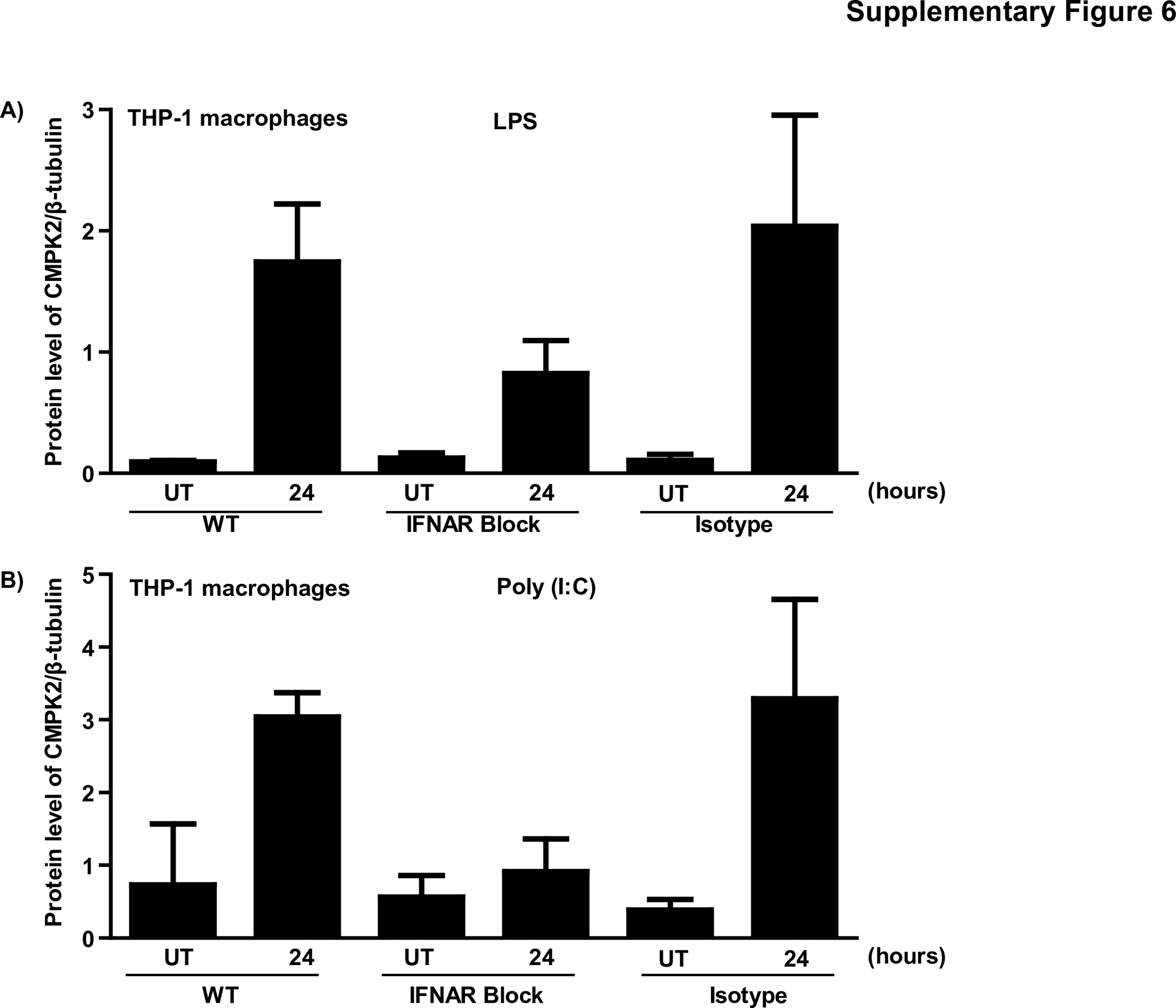

Supplement: S6 Fig — THP-1 cells preincubated with control mAbs (MOPC-173) or IFNAR chain 2 mAbs were stimulated with (A) LPS and (B) Poly (I:C). Following lysates were subjected to western blot analysis. Western blotting results were quantified with ImageJ and presented as the CMPK2/ β-tubulin ratio. Bars represent the mean ± SEM of three independent experiments. (TIF) [file pone.0258989.s006.tif]

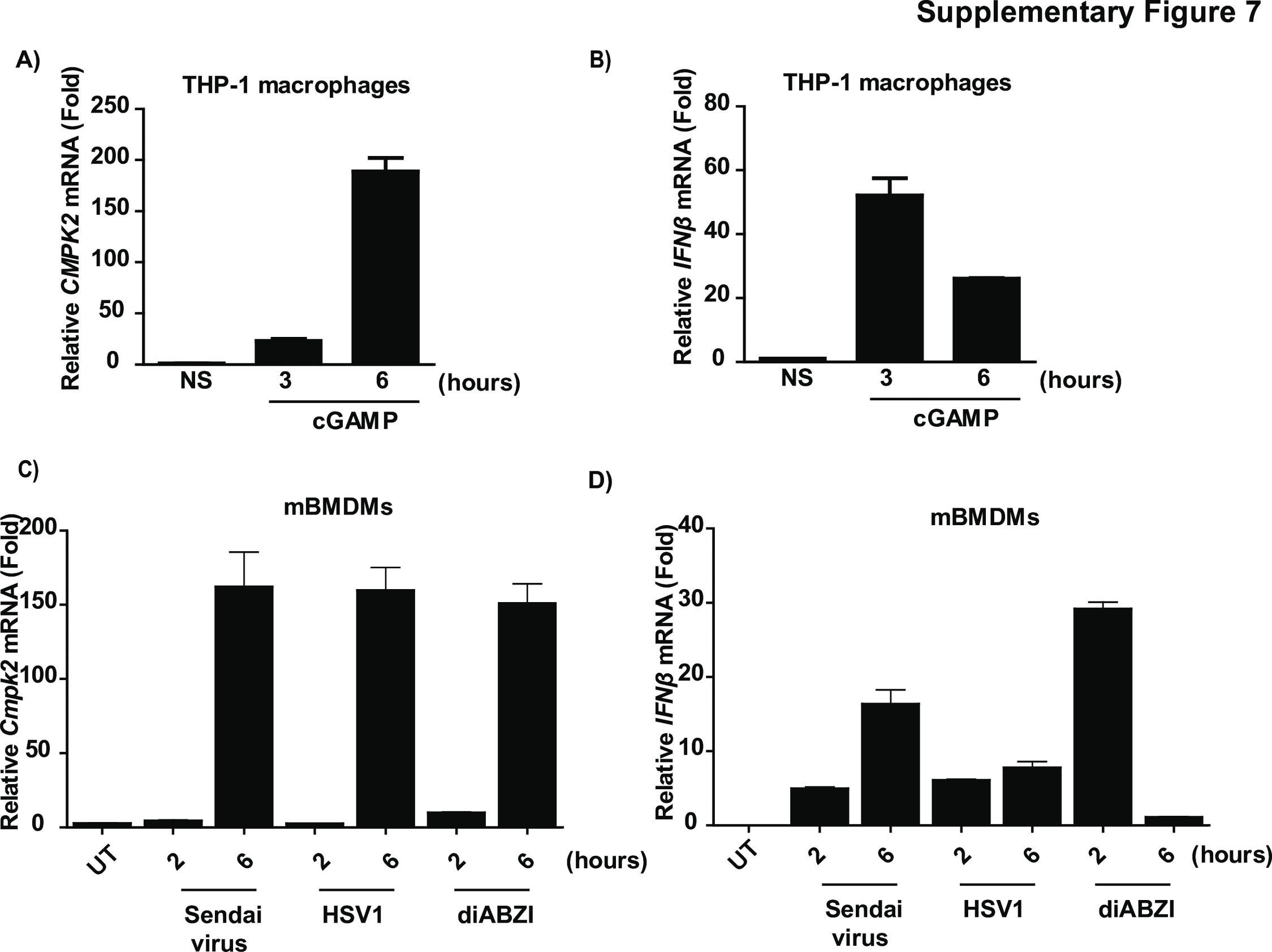

Supplement: S7 Fig — THP-1 cells were transfected with STING ligand, 2’3’-cGAMP (1 ug/ml) using Lipofectamine 3000 then assayed for (A) CMPK2 and (B) IFNβ mRNA expression. Further, mBMDMs were infected with Sendai virus (RIG-1, 10 HA units/ml), Herpes Simplex Virus 1 (HSV1) (cGAS-STING pathway, MOI = 1) and treated with STING agonist–diABZI STING agonist-1 were assayed for (C) CMPK2 and (D) IFNβ mRNA expression. Bars represent the mean ± SEM of three independent experiments. (TIF) [file pone.0258989.s007.tif]
